# EVOLUTION AND EVALUATION OF AUTOLOGOUS MINI PUNCH GRAFTING IN VITILIGO

**DOI:** 10.4103/0019-5154.53195

**Published:** 2009

**Authors:** Koushik Lahiri

**Affiliations:** *From the Department of Dermatology, Apollo Gleneagles Hospital and Pigmentary Disorder Unit, Rita Skin Foundation, India*

**Keywords:** *Mini grafting*, *mini punch grafting*, *punch grafting*, *vitiligo*, *vitiligo surgery*

## Abstract

Vitiligo is a result of disrupted epidermal melanization with an undecided etiology and incompletely understood pathogenesis. Various treatment options have resulted in various degrees of success. Various surgical modalities and transplantation techniques have evolved during the last few decades. Of them, miniature punch grafting (PG) has established its place as the easiest, fastest, and least expensive method. Various aspects of this particular procedure have been discussed here. The historical perspective, the instruments, evolution of mini grafting down the ages, and the methodology, advantages, and disadvantages have been discussed. A detailed discussion on the topic along with a review of relevant literature has been provided in this article.

## Historical Perspective

Vitiligo is the most significant form of cutaneous achromia which is an illusive, if not enigmatic problem down the ages. In all the ancient civilizations and religions there is some reference to vitiligo.

In the Rigveda it was referred to as ‘Kilas’ meaning a white spotted deer. It is interesting to note that as per a Vedic myth the anthropomorphic adoration of the sun, Bhagavantam, developed vitiligo after being gazed upon by his illegitimate son.[1]

The disease was mentioned in ‘Tarkh-e-Tibbl-e-Iran’ in the period of the Aushooriyan, as early as 2200 BC.

In 1550 BC, information regarding vitiligo was noted in the Ebers Papyrus.[2]

Unfortunately in some of the prominent ancient texts the disease was confused with leprosy and also other skin diseases like psoriasis.

This was referred to as ‘Swethakushtha’(white leprosy) in the Atharba Veda (1400 BC).[3]

An accurate description also exists in a collection of Japanese Shinto prayers, Amarakosa, dating from 1200 BC.[4]

In the Old Testament, the white spots were also described in Verse 2 of Chapter 13 of Leviticus under the Hebrew word ‘Zora at’ or ‘Tzaraat/Tzoraath’. Tsoraath was translated using the Greek word “Lepros,” which means a scale or scales. Along with this the phonetic resemblance of the word Tsoraath (which in Ashkenazi pronunciation would read tsoraas) led to the belief that ‘leprosy’ was psoriasis.

In truth, in all likelihood, most cases of biblical ‘leprosy’ were achromic or hypochromic disorders that would include vitiligo, some cases of psoriasis, cases of pityriasis alba, probably albinism, and also leprosy. The word got translated as ‘lepra’ in the Greek and English translations of the Bible. Also the theory of vitiligo as a dirty / polluted / contaminated disease was initiated, as the word ‘Tzaraat’ refers to a group of skin diseases, which, according to the Old Testament, renders one ritually unclean.[5]

The exact word ‘Vitiligo’ may have been derived from the Latin word *vitium* meaning ‘blemish,’ or possibly *vitulum* meaning ‘small blemish.’[5]

Another theory is that the Latin derivation is from the white, glistening flesh of calves (vitelius). Others believe that the actual word was first used by Celsus in his tome De Medicina in the first century A.D.[4,6,7]

References to this disease can also be found in the ancient Koran and Buddhist scripts.[7]

The treatment has undergone an enormous evolutionary change from the Vedic days of ‘Vasuchika’ to the most modern transplantation techniques. However, the ultimate goal remains the same, which is, replenishment of lost pigment.

## Vitiligo Surgery

Many patients respond to standard medical treatment options. However, several patients remain recalcitrant or respond only partially. Any attempt to repigment these resistant patches with conventional medicinal modalities is often unsuccessful and sometimes frustrating, indicating the absence of a melanocytes reservoir, to induce repigmentation. Under these circumstances, melanocyte repopulation of the achromic areas is not possible, unless a new source of pigment cells is placed by surgical methods within the depigmented lesion/s.

Different corrective surgical methods have evolved during the last four decades. Some of these are: Thin Thiersch's graft,[8] epidermal grafting by suction blister,[9] punch grafting,[10] mini punch grafting,[11,12] cultured melanocyte grafting,[13,14] grafting of cultured epidermis,[15] autografting, and PUVA,[16,17] transplantation of autologous cultured melanocytes,[18] single hair transplant,[19] ultrathin epidermal sheets and basal cell layer suspension[20] and mini grafting and NB-UVB.[21]

Among all these, mini punch grafting (PG) is the easiest, fastest, least aggressive, and a technique with minimal expenses.

## The Punch Instrument

The skin punch or surgical punch is an instrument that is used almost exclusively by dermatologists. It is interesting to note that originally it was used as a trephine to cut through the skull bone. Its use was documented in abscess removal from the tibia, as early as, 1852.[22] In 1878 Watson described the use of it in the correction of accidental gunpowder disfigurement.[23]

However, it was E L Keyes, in 1887, who first established the importance of the punch instrument in dermatology.[24]

**Figure 1:**
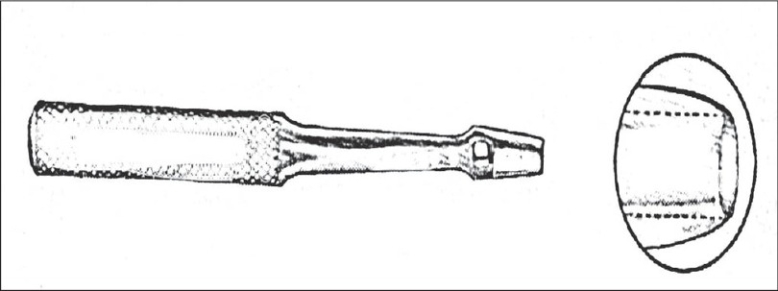
Keye's punch

The Keyes punch [Figure 1] has been used in dermatology since then for diagnostic purposes. Its rounded sharp cutting end and thick handle make it very appropriate for small skin biopsies. Because of the thick walls with angled sides above the cutting edge, the tissue leans to be pushed away as the punch is made, causing less dermis to be cut through (in diameter) than the overlying epidermis. This is also a function of the bevel, which is outside the barrel of the Keyes punch.[25]

To overcome these difficulties other punches have been developed.[26]

**Figure 2:**
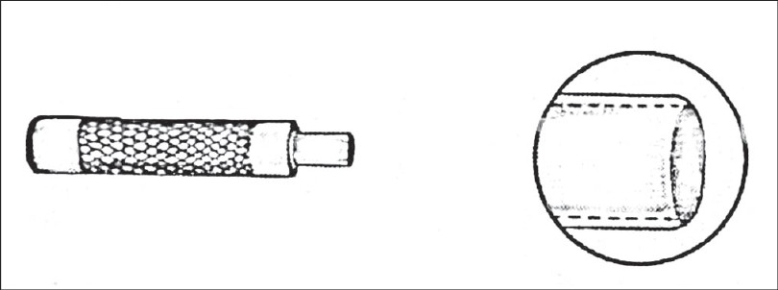
Loo trephine

The walls of the Loo trephine [Figure 2] are thinner and less slanted than those of the Keyes punch, making it advantageous to use on depressed scars or minor autotransplants (where a straight vertical incision is needed) This is difficult to carry out with a Keyes punch.

**Figure 3:**
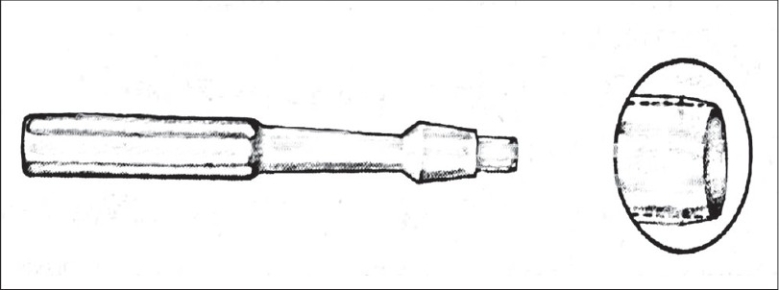
Disposable punch

The newer disposable punches [Figure 3] are excellent for punch biopsies or excisional work on cysts. The razor-sharp edges are a great benefit. The punches are made in a number of different dimensions.

At present, the consensus is toward using miniature punches with smaller diameters for the cutting edge, for vitiligo surgery.

## Evolution of Mini Punch Grafting

In the history of skin grafting a couple of annotations can be referred to as a prelude to further discussion on punch grafting.

The first documented successful result of experimental skin grafting was described in sheep by Baronio in 1804.[27]

However, it took almost another one-and-a-half centuries to get the first recorded autograft response of dark-skinned autografts when transplanted to light areas in spotted guinea pigs, by Lewin and Peck in 1941.[28]

In 1972, Norman Orentriech first reported autograft repigmentation in humans. He treated a black woman with longstanding leucoderma, which followed a chemical burn many years back, when she was treated with a home remedy that included a copper penny dipped in vinegar for presumptive tinea infection on her cheek. Orentriech deployed nine, 1 and 2mm diameter, normal skin autografts and observed the ‘pigment spread phenomenon’. He reported a maximum of 1 mm pigment spread from both the 1 and 2mm grafts.[10]

In 1976, Labuono and Shatin made a similar observation after transplanting hair bulbs with hair punch grafting within the leucodermic scars of discoid lupus erythematosus (DLE).[29]

Falabella, in 1978, reported a novel method of repigmenting leucoderma. With the help of a power-driven dermabrasion unit, he used dental burrs to create abrasions, 2-3mm in diameter, less than 1mm in depth, and 5mm apart. In the donor area, the skin was raised by means of a curved needle, and was sniffed off just below it, to harvest 1-2mm size minigrafts. He reported about a 3mm perigraft pigment spread by this technique. Three patients, one with piebaldism, another with chemical leucoderma and a third with post-burn depigmentation were treated by this method.[30] He observed that these superficial split thickness grafts evoked a much better outcome than the full thickness hair punch grafting performed by Labuono.

Interestingly, in the same article, it was concluded that ‘true vitiligo is not treatable by transplantation of grafts of normally pigmented, autologous skin’.[30]

Miniature punches on a 1.5mm diameter were used by Falabella for repigmenting three cases of segmental vitiligo in 1983.[31]

Behl expressed some reservation while commenting on Falabella's work on mini grafting and claimed better results with thin Thiersch grafting. In a rejoinder Falabella reiterated his faith in miniature punch grafting and countered with his sets of reasons and logics in favor of punch grafting.[32,33]

In the following years Falabella reported success with mini punch grafting in chemical leucoderma, post-dermabrasion leucoderma, and focal and segmental vitiligo.[34–36]

When repigmenting stable leucoderma with autologous mini grafting, Falabella made an important observation with regard to the relationship between the donor graft area and the area of surgical repigmentation, and found that a 1mm donor graft could originate a pigmented spot 25 times larger than its size.[34]

In 1995, it was Falabella again who combined epidermal grafting and mini grafting in treating vitiligo and piebaldism.[37]

Westerhof, in 1994, reported success with punch grafting in stable vitiligo and observed a maximum of 5mm of pigment spread.[38] In the subsequent year (1995), Boersma stressed on the importance of a proper selection of cases before mini grafting.[39]

Various studies point toward the high effectiveness of the procedure.[40–43] An assortment of different evaluation parameters of mini punch grafting have also evolved over the years.[44–47]

It has now been documented that mini punch grafting in combination with NB-UVB (311nm) and has shown encouraging results.[21]

## Test Grafting

Before embarking upon any surgical intervention in vitiligo, a proper assessment of the stability status is of paramount importance. In recent times, this concept has been discussed in detail.[48]

Clinically, stability can be judged by three simple indicators:

- History
- Lack of progression of old lesions and absence of development of any new lesion within a specified period (6 months to 2 years)
- Koebner phenomenon (Kp)
- Absence of a recent Kp either from history (Kp-h) or experimentally induced (Kp-e)
- Test grafting

On the backdrop of persistent incongruity about the minimal period of stability, an attempt was made for the first time by Falabella, in 1995, to fathom the stability before surgery, by introducing a mini grafting test.[49]

The objective of this test was to:

- Establish the stability of the depigmenting process
- Determine a means by which patients could be selected
- Identify patients who may respond to pigment cell transplantation
- Anticipate the response to surgical repair

In the original suggested procedure a few grafts (1.0-1.2mm) were placed in the center of the depigmented lesion to be scrutinized. Dressing was done by Micorpore^®^ adhesive tape and kept for a couple of weeks. After removal of the tape the area was exposed to sunlight for 15 minutes daily, for a period of 3 months. No treatment was permitted during this test period.

All test sites were visualized under Wood's light. The test was considered positive if unequivocal repigmentation took place beyond 1 mm from the border of the implanted grafts. On the other hand, if less than 1 mm or no repigmentation was observed the test was considered to be negative.

In some of the biggest series, this evaluation has been termed as ‘test grafting’ (TG) and is found to be a more reliable exercise than the unjustified dependence on the period of stability alone.[21,43,50]

Over the years this ‘test’ has been vindicated and acknowledged as a powerful tool for detecting stable vitiligo, which anticipates the repigmentation success in vitiligo when surgery becomes a therapeutic option.

## The Method of Mini Grafting

After proper assessment of the stability status and routine physical examination and investigations, an informed consent is taken from the patient, and the donor and recipient areas are surgically prepared.

1. The instruments required are 1.5 or 1.2mm punches, small jeweler's or graft holding forceps, and a small curved tip scissors.
2. The recipient area is prepared first. Two percent lignocaine with or without adrenaline is infiltrated as a local anesthetic.
3. To minimize the chance of developing any perigraft halo, the initial recipient chambers are made on or very close to the border of the lesion. The punched out chambers are spaced according to the result of test grafting or at a gap of 5-10mm from each other.
4. The donor area is either the upper lateral portion of the thigh or the gluteal area. Punch impressions are made very close to each other so that a maximum number of grafts can be taken from a small area.
5. Same sized punches are used for both the donor and recipient areas.
6. The grafts are placed directly from the donor (buttock/upper thigh) to the recipient areas. This speeds up the procedure and lessens the chance of infection. Care is taken to ensure that the graft edges are not folded and the tissue is not crushed or placed upside down. The needle of the syringe or the tip of the scissors is used for the proper placement of grafts in the recipient chambers.
7. Hemostasis is achieved by pressing a saline-soaked gauze piece over the area.
8. For the recipient area, the three layers of dressing from inside to out are: Paraffin-embedded, nonadherent sterile gauze (Jelonet^®^), sterile Surgipad^®^, and bio-occlusive Micropore^®^.
9. For the donor area only Surgipad^®^ and Micropore^®^ are used.
10. The recipient area may be immobilized if necessary. Proper instructions for special areas like the lips are necessary. To secure the recipient area these patients are advised to be on a liquid diet for the first 24 hours, preferably with a straw. Patients are allowed a normal diet after this period.
11. Sometimes dressings are opened after 24 hours to look for any dislodgement of grafts, if any are found, they are replaced.
12. Finally after 4 to 7 days the dressings are removed.[31,41–43]

## Follow-up and Course of Events

Post-surgically the patients are exposed to PUVA[16,17]/PUVASOL (Psoralen plus UVA from Sunlight)[42,43] or NB- UVB[21] or even kept as such in some studies.[43] The patients are followed up fortnightly for the initial two months and then monthly, until complete repigmentation is achieved [Figures 4–12].

In the donor site, after healing with secondary intention, minimal superficial scarring is expected and acceptable.

Scabs may fall off from the recipient site within 7-14 days. However, in many instances there may not be any scab formation. Perigraft repigmentation is expected to start from around 3-4 weeks.[21,42–43]

The entire depigmented and grafted area is expected to be completely repigmenetd within 3-6 months, based on the area of grafting and the body part involved.

**Figure 4:**
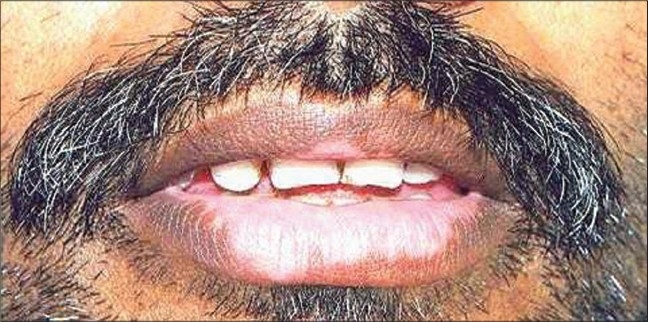
Lip vitiligo

**Figure 5:**
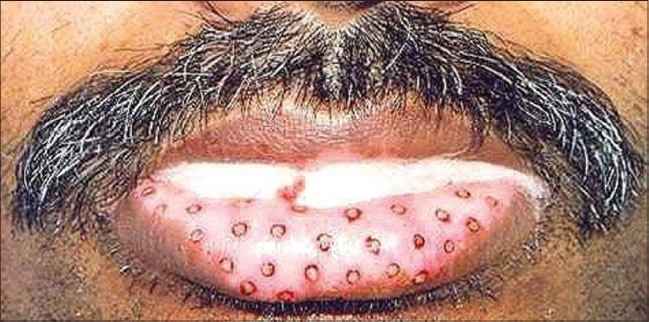
Correct positioning of graft

**Figure 6:**
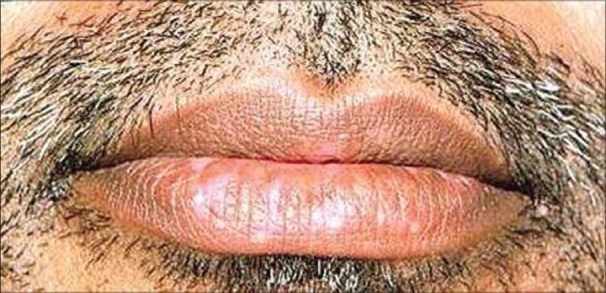
Excellent repigmentation after 9 months

**Figure 7:**
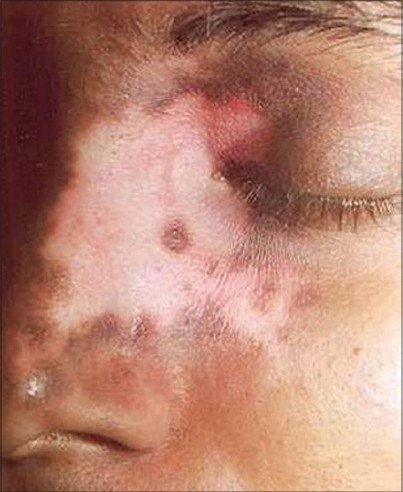
Segmental vitiligo

**Figure 8:**
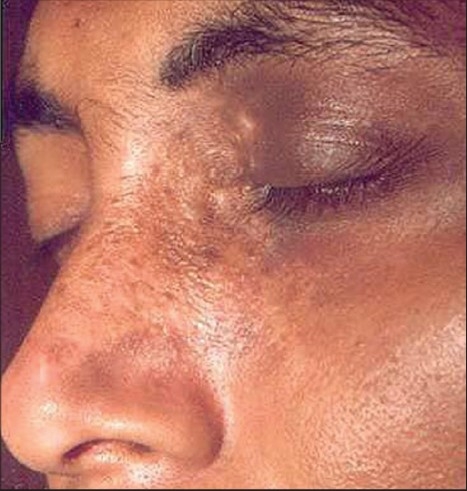
Complete repigmentation after 6 months

**Figure 9:**
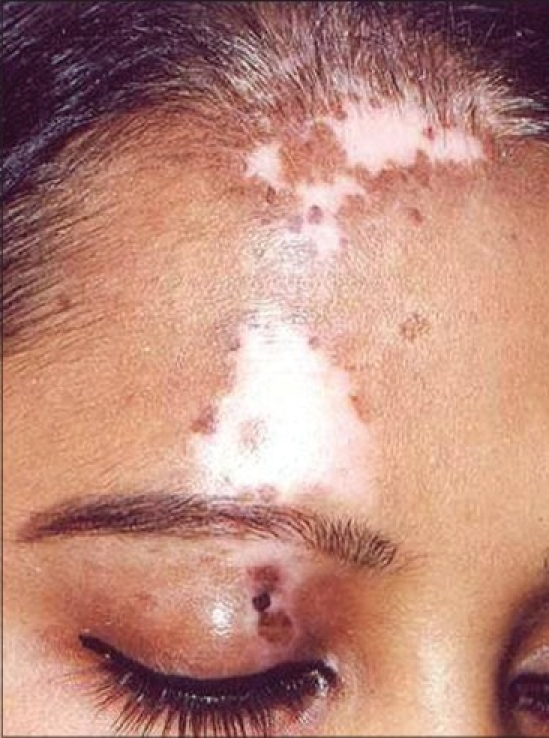
Segmental vitiligo

**Figure 10:**
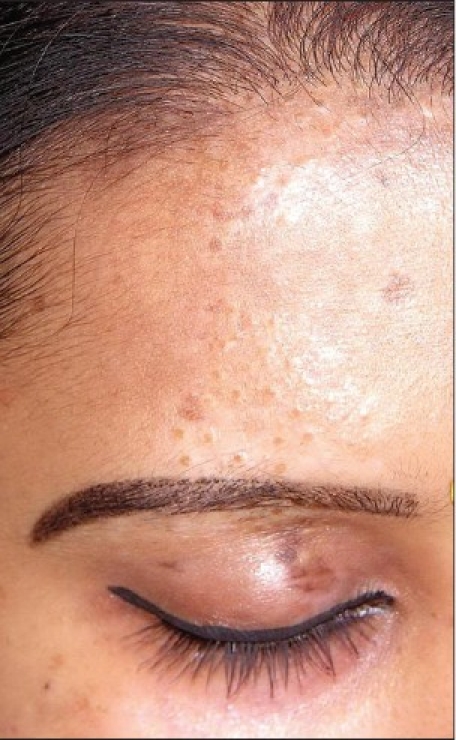
Complete repigmentation with repigmentation of leucotrichia after 5 months

**Figure 11:**
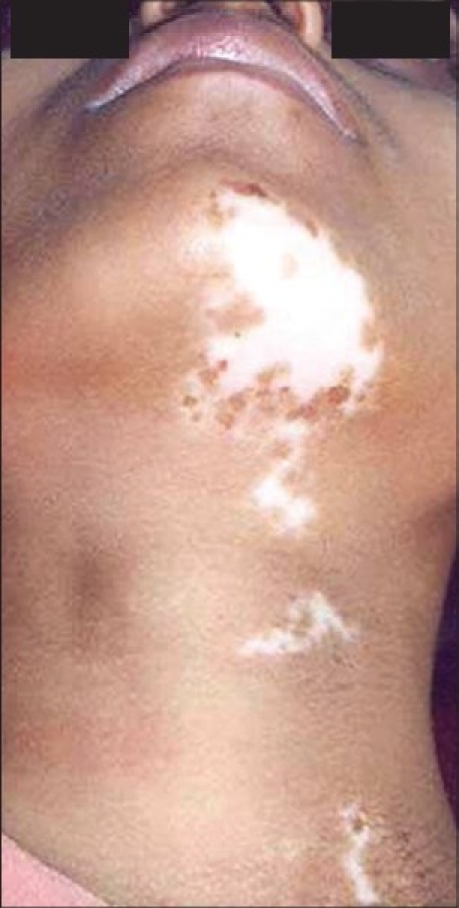
Segmental vitiligo on neck

**Figure 12:**
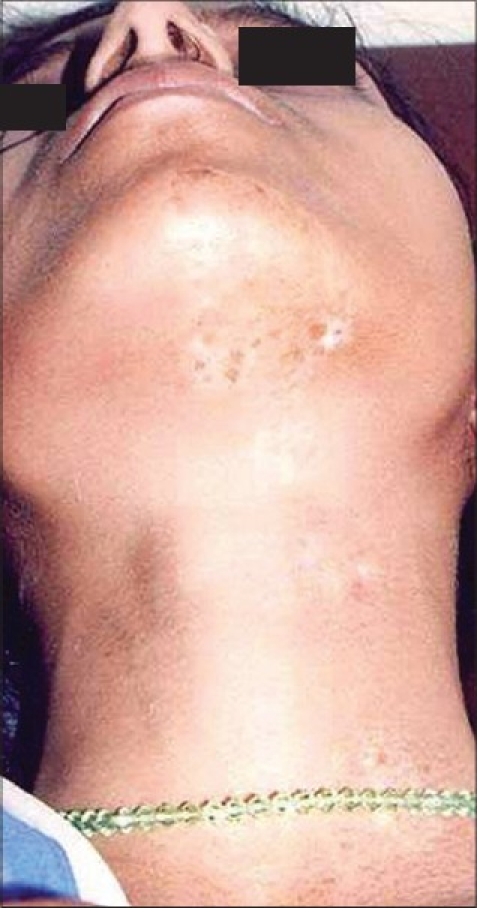
Complete repigmentation after 8 months

## Complications

### Recipient site

1. Cobble stoning
2. Polka dot
3. Variegated appearance and color mismatch
4. Static graft (no pigment spread)
5. Depigmentation of graft
6. Perigraft halo
7. Graft dislodgement / rejection
8. Hypertrophic scar and Keloid formation

### Donor site

1. Keloid
2. Hypertrophic scar
3. Superficial scar
4. Depigmentation / spread of disease
5. Contact dermatitis to adhesive tapes

By proper selection of cases most of these complications are entirely avoidable.[31,41–43]

Cobble stoning is regarded as the commonest of them all.[21,42,43,50]

It was observed that with time it got corrected in most of the cases.[42] In resistant cases corrective electrofulguration may be needed.[51]

In this regard it is only apt to conclude that grafting should not be performed with punches more than 1.5mm in diameter. On face and lips it should be even smaller (1.2mm or 1mm).[21,52]

Herpes labialis-induced lip leucoderma (HILL) is another unpredictable entity bearing the risk of rejection of grafts.[53–56]

### Advantages

1. Easiest, fastest, and least expensive method
2. High rate of success with very few preventable / manageable side effects
3. Can be performed anywhere, on any site (except angle of the mouth)

## Discussion

Surgical correction of vitiligo and other cutaneous achromia has come a long way in the last almost five decades.

However, among all other methods, autologous miniature punch grafting has established its place as the easiest, fastest, safest, and least aggressive means of vitiligo surgery.

When the graft is taken off, the piece of tissue is completely detached from the donor site and then placed on the vascular bed in the recipient holes. From this vascular bed it derives its blood supply. Initially the graft adheres to its new bed with the help of fibrin. There is diffusion of nutrients through this fibrinous layer, which keeps the graft alive initially. Within 2-3 days, capillary linkage occurs, with vascularization of the graft. The thinner the graft the denser the capillary network in the superficial dermis, and thus earlier is the process of vascularization.[57]

Phototherapy-induced stimulation of melanocyte migration from the hair follicle reservoir is now a well-established fact. It spreads centrifugally from the infundibulum to the basal cell layer and recolonizes the epidermis with active and functional melanocytes.[58,59] However, the presence of a pilosebaceous apparatus within the mini grafts is not at all necessary for the repigmentation process, as in suction blister grafts only the epithelial cells present in the grafts are sufficient to induce repigmentation.[60] In 1970, Billingham and Silvers have demonstrated the phenomenon of melanocyte migration from the graft's edge, within the achromic skin, to recolonize and replenish the area with functional and active melanocytes.[61]

Falabella, in 1988, tried to establish a histological / histochemical background of surgical repigmentation.[36]

In another study a consistent and comparable status of melanization was noted over both normal and surgically repigmented areas by using the Masson Fontana stain.[45] Even after recognizing the significance of stability, and after three decades of experience in vitiligo surgery, it is quite incongruous to note the little consensus regarding the optimal required period of stability. The complete lack of unanimity can be glaring in some instances [Table 1]. In one study the minimal period of stability, as a prerequisite for grafting, was mentioned to be as little as a period of 4 months.[62] While on the other side of the spectrum, in another study, it was taken as 3 years.[20,21]

Other variable figures like 6 months, 1 year, and 2 years can easily be obtained from some other studies as well.[39,40,63]

Even the same author has taken different periods of stability into consideration in different articles.[15,33]

Recently, in their consensus recommendations, the IADVL Task Force for standard guidelines of care for dermatosurgical procedures tried to provide a clear definition of stability as ‘a patient reporting no new lesions, no progression of existing lesions, and absence of Koebner phenomenon during the past one year’.[74]

It is often difficult to predict how long the disease will remain stable. Similarly, it is difficult to envisage when it will start becoming unstable.[64,65]

Repigmentation has been successfully induced in previous graft failure cases under NB-UVB (311 nm) phototherapy.[66]

The observation of spontaneous repigmentation of non-grafted vitiligo patches points toward a possible release of fresh cytokines from the donor skin, although stimulating the vitiliginous patches and hair follicles of the grafted sites may have played some role at the distant sites by local absorption.[67,68] Another theory is that the immunogenic mechanism, which was originally responsible for the development of vitiligo, may have lost its antigenicity due to the autologous grafts.[69]

The size of the grafted lesion varied between 15 to 144 cm^2^ in different studies [Table 2]. Likewise the size of the punch instrument differed in different studies [Table 3]. However, now the consensus is toward using smaller punches, 1.2 or 1.5mm. Falabella even recommends 1mm grafts for the facial region and 1.2mm for other body parts.[52]

In this way the commonest complication of punch grafting can also be avoided. Although the rate of cobblestoning was substantial in most of the study, it was found that with time it got corrected. In resistant cases electrofulguration was helpful.[70]

Very recently repigmentation of leucotrichia with PG and NB-UVB was reported.[21] The same was also observed and documented before, with PUVASOL and PG.[71]

Another important parameter was the post-graft appearance of repigmentation (AOR) time. It was found to be between 2 and 6 weeks, in different studies [Table 4].

After PG and PUVASOL (Psoralen plus ultraviolet A from solar radiation), the appearance of repigmentation (AOR) time in different regions varied between 14 and 39 days, with an overall average being approximately 21.6 days, as shown in one study.[42] With the deployment of NB-UVB along with PG, the appearance of repigmentation (AOR) time in different regions varied between 14 and 32 days, with an overall average being approximately 20.6 days.[21]

Orentriech in his original article observed that irrespective of whether 1 or 2mm grafts were employed, the pigment spread was consistently 1mm.[10]

Various other results can be found in the literature [Table 5].

Falabella, while establishing a relationship between donor graft and the area of surgical repigmentation, found that a 1mm donor graft could repigment an area 25 times larger than the graft itself.[34] In a recent study with 1.5mm grafts and NB-UVB, in subjects with skin types IV and V, this value was found to be more than double of that (56.21 times).[21] Previously in one study with PUVASOL this relationship was found to be 42 times the size of the graft.[47] Bigger grafts, darker skin types, and deployment of NB phototherapy could all have accounted for this high statistical value. Considering all the indicators and parameters, mini grafting is not only the easiest, safest, and least expensive method, but it is one of the most effective treatment options in treating stable and recalcitrant vitiligo.

**Table 1:** Minimum period of stability in different studies

Author^Ref^	Year	Period of stability
Das SS, Pasricha JS[62]	1992	4 months
Boersma BR, Westerhof W[39]	1995	6 months
Jha AK, Pandey SS, Shukla VK[63]	1992	1 year
Savant SS[40]	1992	2 years
Falabella R[33]	1995	2 years
Falabella R[15]	1992	3 years

**Table 2:** Maximum grafted area in different studies

Author^Ref^	Year	Maximum grafted area (cm^2^)
Jha AK, Pandey SS, Shukla VK[63]	1992	15
Das S, Pasricha JS[62]	1992	80
Boersma BR, Westerhof W[62]	1995	96
Malakar S, Dhar S[43]	1999	>100
Falabella R[36]	1988	110
Lahiri K, Sengupta SR[42]	1997	144

**Table 3:** Size of graft in different studies

Author^Ref^	Year	Size of punch (diameter in mm)
Das S, Pasricha JS[62]	1992	4
Jha AK, Pandey SS, Shukla VK[63]	1992	3 and 4
Boersma BR, Westerhof W[39]	1995	2
Lahiri K, Sengupta SR[42]	1997	2
Malakar S, Dhar S[43]	1999	2
Malakar S, Lahiri K[50]	2004	1.5
Falabella R[36]	1988	1.2
Orentriech N, Selmanwitz VJ[10]	1972	1 and 2

**Table 4:** Appearance of repigmentation time in different studies

Author^Ref^	Year	Earliest AOR (in days)
Lahiri K, Malakar S *et al*.[21]	2005	14
Lahiri K, Sengupta SR[42]	1997	14
Malakar S, Dhar S[43]	1999	16
Savant SS[40]	1992	30

**Table 5:** Maximum pigment spread in different studies

Author^Ref^	Year	MPS (in mm)
Orentriech N, Selmanwitz VJ[10]	1972	1
Falabella R[30]	1978	3
Falabella R[36]	1988	4
Westerhof W, Boersma M[38]	1994	5
Savant SS[40]	1992	15
Lahiri K, Sengupta SR[42]	1997	10
Malakar S, Dhar S[43]	1999	10
Lahiri K, Malakar S *et al*.[21]	2005	12

MPS: Maximum pigment spread
